# Associations between Parental Protective Factors and Child Behavioral Problems in Children with ADHD and ASD

**DOI:** 10.1007/s10882-025-10018-8

**Published:** 2025-05-06

**Authors:** Charlotte Verhagen, Myrthe Boekhorst, Nina Kupper, Franca Leeuwis, Stefanie Duijndam

**Affiliations:** 1 Department of Medical and Clinical Psychology CoRPS – Center of Research on Psychological Disorders and Somatic Diseases, Tilburg School of Social and Behavioral Sciences, Tilburg University, PO box 90153, Tilburg, 5000LE The Netherlands; 2 VU Amsterdam, Amsterdam, The Netherlands

**Keywords:** Parental resilience, Parental empowerment, Child behavioral problems, Neurodivergence, Parenting

## Abstract

Previous research in neurodivergent children has shown a relation between parental risk factors and child internalizing and externalizing behaviors. Yet, a paucity of studies has examined the association between parental protective factors and child outcomes. This study investigated the association between parental empowerment and resilience and the degree of internalizing and externalizing behaviors in children with autism spectrum disorder (ASD) and attention deficit hyperactivity disorder (ADHD). Data were collected through the the In Kaart register between September 2022 and February 2024. Children aged 5 to 15 years (*M* = 11.1, 32.9% female) with a diagnosis of ADHD and/or ASD were included. Parents (97.1% mothers) filled in questionnaires about their levels of resilience and empowerment, and about their children’s behavioral problems. Hierarchical regression analyses revealed that younger child age and higher levels of parental resilience were associated with more externalizing behaviors. Child age did not significantly moderate the relation between resilience and externalizing behaviors. Nevertheless, the pattern observed in the data suggested potential age-related differences in how parental resilience is associated with child behavior. The preliminary findings suggest that resilience might be a mechanism for adapting to increased parenting demands associated with raising a neurodivergent child with problem behaviors. Furthermore, parental empowerment may not be directly associated with child problems, giving room for future research to delve into other factors that play a role in the association between parental protective factors and child outcomes. The current findings highlight the need to examine this relation in larger, more diverse samples.

## Associations between Parental Protective Factors and Child Behavioral Problem in Children with ADHD and ASD

Neurodivergent conditions are prevalent among children and are thought to affect 15–20% of children aged 3 to 17 years (Francés et al., 2022). The two most common neurodivergent conditions are autism spectrum disorder (ASD) and attention deficit and hyperactivity disorder (ADHD). According to the DSM-V, ASD is defined by impairments in social communication and interaction, and restricted and repetitive behavior, interests, and activities (APA, 2013). ADHD on the other hand, is characterized by inattention and/or hyperactivity-impulsivity, negatively impacting psychosocial functioning (APA, 2013). In children with neurodivergence, core symptoms often co-occur with internalizing and externalizing symptoms (McRae et al., 2020; Operto et al., 2021). Whereas internalizing problem behavior is directed inwards (e.g., anxiety, depression), externalizing behavior specifically occurs in interaction with the social environment (e.g., aggression, deviance). High prevalence of these symptoms in neurodivergent children has been associated with greater peer problems and poorer quality of life (Andersen et al., 2023; Armstrong et al., 2015). Thus, understanding the factors influencing the presence of internalizing and externalizing problems in these children is important.

Findings have indicated several child factors associated with a higher presence of internalizing and externalizing problems in neurodivergent children, including lower cognitive and socialization skills in ASD, and symptom severity in ADHD (Donoso et al., 2023; Mlodnicka et al., 2024). Furthermore, an extensive body of research has investigated parental factors that are associated with behavioral problems in neurodivergent children (e.g., McRae et al., 2019). Most studies have focused on the impact of parental risk factors on child behavior, such as parenting stress (i.e., the aversive psychological reaction to the demands of being a parent). In children with ASD and ADHD, findings have indicated that higher levels of parenting stress were positively linked to the occurrence of emotional and behavioral problems (Gordon & Hinshaw, 2017; McRae et al., 2020).

On the other hand, literature suggests that positive parental factors might be associated with fewer behavioral and emotional problems in children with ASD and ADHD (Cabrera et al., 2021). Some protective factors that have recently gained more attention are parental resilience and empowerment (Damen et al., 2017; Gavidia-Payne et al., 2015). Parental resilience is defined as the capacity of parents to deliver a competent and quality level of parenting despite the presence of risk factors (Gavidia-Payne et al., 2015). Resilience is considered more of a process than a trait and can therefore vary over time, strengthening with experience. Several parental factors are associated with resilient parenting outcomes, including adaptive coping styles, social support, and psychological well-being (Cantero-Garcia & Alonso-Tapia, 2018; Gavidia-Payne et al., 2015; McConnell et al., 2014). These latter traits in turn were associated with less internalizing and externalizing symptoms in neurotypical children (Clayborne et al., 2023). Next, parental empowerment is defined as the parenting competency to address and solve parenting problems and make the right decisions regarding parenting issues (Damen et al., 2017). By strengthening feelings of empowerment, parents increase their feelings of personal control, their critical awareness of handling parenting issues, and their parental control over their child (Damen et al., 2021). Consequently, research has indicated that parental empowerment seems to be related to fewer behavioral problems in youth (Damen et al., 2019).

These previous studies have mainly focused on neurotypical children, while there is a lack of research investigating the association between these parental protective factors and child socio-emotional outcomes in neurodivergent children. With respect to resilience, research suggests that it plays a role in predicting the quality of life in children with developmental disabilities, including ASD and ADHD (Widyawati et al., 2023). In addition, the findings of Song et al. (2021) showed that lower family resilience was associated with more conduct and mental health problems in children with ADHD. Parental empowerment, on the other hand, has been merely examined as a mediator in the positive relation between maternal distress and child aggression in children with ASD (Weiss et al., 2015). Thus, there seems to be an absence of studies examining the direct associations between parental resilience and empowerment and socio-emotional outcomes in both children with ASD and ADHD. These findings suggest that resilience and empowerment might buffer the negative effects of parental stress on behavioral problems in children with ASD and ADHD. Higher levels of resilience and empowerment may promote the use of adaptive coping strategies to address stressful situations and improve perceptions of challenging child behaviors, which are prevalent in children with ASD and ADHD (Clayborne et al., 2023; Damen et al., 2021). Consequently, higher levels of these protective factors may contribute to parents’ ability to perform nurturing parenting, such as being warm, responsive and sensitive to children’s needs and behaviors. In turn, nurturing parenting has been shown to be associated with fewer internalizing and externalizing behavior problems in ASD and ADHD (McRae et al., 2019).

Thus, parental protective-and risk factors seem to affect parenting behaviors, which in turn, are associated with internalizing and externalizing problems in children with ASD and ADHD. While extensive research has been conducted on parental risk factors, such as parental distress, there has been very little focus on parental protective factors, especially in parents of children with ASD and ADHD. Investigating these factors can inform parenting programs and interventions to promote parental resilience and empowerment, with the goal of enhancing parental mental health, improving parenting quality, and reducing the risk of behavioral problems in children with ASD and ADHD.

There is a need for research that examines whether positive parenting factors are related to child adjustment in neurodivergent children. Therefore, this study will investigate the association between parental feelings of empowerment and resilience and the degree of internalizing and externalizing behaviors in children with ASD and/or ADHD. It is hypothesized that feelings of resilience and empowerment will be negatively associated with the presence of externalizing and internalizing symptoms in these children.

## Materials and Methods

### Participants

The current study included 70 neurodivergent children and their parents, who are part of the the In Kaart register. In Kaart has been developed in collaboration with the target group, with the ambition to better the quality of lives of people with neurodivergence and/or disabilities and their families and to gain more knowledge on the differences and similarities of people with neurodivergence and/or disabilities. In Kaart is an open cohort study that collects data from people (age 0–99 years) who are neurodivergent and/or have disabilities in the Netherlands. Participants who have a (suspected) diagnosis of ASD, ADHD, developmental co-ordination disorder (DCD), dyslexia, developmental language disorder (DLD), visual impairment and intellectual disability, or have a child with the abovementioned (suspected) diagnoses, are eligible for participation. For the current study, only parents of children aged below 16 years with a diagnosis of ASD and/or ADHD, will be included, aligning with the study’s focus on the child–and early adolescence population. To verify diagnoses, participants were asked to provide additional information, including the child’s age at diagnosis, the professional who made the diagnosis, and the location where it was made. Participants with merely a suspected diagnosis of ADHD or ASD are excluded from analysis. The final study sample included 70 children aged 5 to 15 years old (*M* = 11.1, *SD* = 2.5) and their parents (M_age_ = 44.9, 97.1% mothers). Most children lived with both parents (70%) and had brother(s) or sister(s) (85.7%).

### Procedure

The data for this study were obtained from In Kaart. Participants were recruited through convenience sampling. In Kaart collaborates closely with several interest groups, including parent organizations, to facilitate participant recruitment. Participants enroll through the In Kaartwebsite (https://www.inkaart.org/information_for_researchers/information-for-researchers) where they complete an informed consent form online. After giving written consent, they received a link to a baseline online questionnaire via email and are additionally invited annually to complete a follow-up questionnaire. For this study, baseline data from parents reporting on their child who are < 16 years old with ADHD and/or ASD were included. These data were combined with parent-report questionnaires as part of a collaborative project (Ouders in Evenwicht), also collected through In Kaart. This project focuses on resilience and burn-out in parents of children with special needs, including children with ADHD and/or ASD. The data used in the current study were collected between September 2022 and February 2024.

Prior to the start of In Kaart, the Medical Ethics Review Committee of the Medical Ethics Review Committee of Amsterdam University Medical Center location VUmc confirmed that the Medical Research Involving Human Subjects Act (WMO) does not apply to In Kaart (no. 2022.0258). Furthermore, In Kaart was reviewed and approved by the Permanent Committee on Science and Ethics (VCWE) of the Faculty of Behavioural and Movement sciences of Vrije Universiteit Amsterdam (no. VCWE-2022-103).

### Measures

#### Family Empowerment Scale (FES)

The family empowerment scale (FES) is a 34-item questionnaire developed by Koren (1992). The questionnaire was created to assess empowerment in parents and other family caretakers of children with emotional disabilities. Each item has five response options, ranging from “not true at all” to “very true”, with higher scores indicating higher levels of empowerment. The items can be divided into three subscales: Family (12 items), Service System (12 items) and Community (10 items). Family refers to the level of empowerment within the home situation, regarding management of daily situations (e.g., “I feel my family life is under control”). Service System refers to parental feelings of empowerment with regards to obtaining adequate services for their children (e.g., “I tell professionals what I think about services being provided to my child”). The Community subscale contains items regarding parents’ involvement in the community and parent advocacy to legislators and policymakers. This last subscale was not administered in the current study. Because the items comprising the Service System subscale showed a non-response rate of around 20%, only the Family subscale was included in the analyses. Previous research shows high reliability and good validity of the family subscale of the FES in Turkish and American samples (Boztepe et al., 2022; Koren, 1992). Likewise, reliability in the current study has been found to be good, with Cronbach’s α = 0.83.

#### Brief Resilience Scale (BRS)

The brief resilience scale (BRS) is a 6-item questionnaire developed by Smith et al. (2008). The short questionnaire was created to measure the ability to bounce back or recover from stress. Each item (e.g., “I tend to bounce back quickly after hard times”) has five response options, ranging from “strongly disagree” to “strongly agree”. Total scores on this scale range from 6 to 30, with higher scores indicating higher resilience. Previous research in American and Dutch samples indicated good reliability of the BRS (Smith et al., 2008; Soer et al., 2019). In the current study, reliability was good, with Cronbach’s α =.83.

#### Strengths and Difficulties Questionnaire (SDQ)

The strengths and difficulties questionnaire (SDQ) is a screening questionnaire for children and adolescents, developed by Goodman (1997). The current study employed the parent-report measure of the SDQ for children aged between 2 and 17 years of age. The 25 items are divided between 5 subscales of 5 items each: emotional symptoms (e.g., “Many fears, easily scared”), conduct problems (e.g., “Steals from home, school or elsewhere”), hyperactivity/inattention (e.g., “Poor concentration or being easily distracted”), peer relationship problems (e.g., “Picked on or bullied by other children”), and prosocial behavior (e.g., “Considerate of other people’s feelings”). The current study included only the first four subscales. Each item has three response options, “not true”, “somewhat true” and “certainly true”. The score for “internalizing problems” is calculated as the sum of the emotional problems scale and the peer problems scale and the score for “externalizing problems” is calculated as the sum of the conduct problems scale and the hyperactivity scale. The SDQ was validated in a Dutch community sample, with McDonald’s omegas (ω) ranging from 0.67 to 0.90 for the parent-report of the five subscales (Stone et al., 2015). In the current study, reliability was good, with ω = 0.81 for internalizing problems and ω = 0.83 for externalizing problems.

### Statistical Analysis

Analyses were completed in Statistical Package for the Social Sciences (IBM SPSS version 28.0). First, outliers were checked by means of histograms and a missing value analysis was conducted. Missing value rates were as follows: 1.4% for the SDQ, 5.7% for the FES, and 4.3% for the BRS. The Little’s Missing Completely at Random (MCAR) Test demonstrated that the data were missing completely at random and unrelated to other variables in the dataset (*χ*^2^ = 163,326, *p* =.544). Based on these results, the use of listwise deletion as a safe option for handling missing data was justified. Then, descriptive statistics and Pearson’s bivariate correlations were calculated between parental resilience, empowerment, child outcomes, and covariates.

For the main analyses, two hierarchical linear regressions were conducted, with the only difference being the outcome variable, namely externalizing problems or internalizing problems. First, parental resilience and empowerment were entered into the model to assess their contributions in explaining child outcomes. For the second model, the covariates (i.e., child age, child sex, and parental education level) were added. Parental education level was recoded into a dichotomous variable comprising low education level (i.e., high school graduate or vocational education) and high education level (i.e., bachelor’s degree or higher), with the former as a reference. A significance level of *p* <.05 was used to determine the statistical significance of the predictor variables. A power analysis was conducted in G*Power with parental empowerment, parental resilience, child age, child sex, and parental education level as predictors and externalizing and internalizing symptoms as outcomes. A power of 0.80 was chosen to maximize the likelihood of detecting significant effects while considering the limited sample size. With a significance criterion of α = 0.05 and a small to medium effect size of f^2^ = 0.15, the required sample size was 92.

The assumptions of normality, linearity, homoscedasticity and multicollinearity were assessed to ensure the validity of the regression analyses. The normality assumption was assessed using the Shapiro-Wilk test, indicating that both resilience (*p* =.04) and empowerment (*p* =.02) were not normally distributed, thus violating this assumption. Nevertheless, the values for both skewness and kurtosis fell within the reasonable range of − 1 to 1 and − 2 to 2, respectively (George & Mallery, 2013). The assumptions for homoscedasticity, multicollinearity and linearity were met.

As an exploratory analysis, if one of the predictors and one or more covariates were significantly associated with the outcome variables, post-hoc moderation analyses were conducted with PROCESS macro v4.2. In addition, we did a sensitivity analysis using the Kruskal-Wallis test, children with ADHD, ASD and with both diagnoses were compared on levels of parental resilience, parental empowerment, internalizing, and externalizing symptoms.

## Results

### Preliminary Analyses

Demographical characteristics of the sample (*N* = 70) and descriptives of the study variables can be found in Table 1 (Table 1). In total, 14 (20.0%) had an ADHD diagnosis only, 39 (55.7%) had an ASD diagnosis only, and 17 (24.3%) had both diagnoses.

**Table 1:** Demographical characteristics and descriptives of the study variables (*N* = 70)

	*N*	%	M (SD)	Range
**Demographics**				
Parent age			44.9 (5.7)	32.3–56.8
Child age			11.1 (2.5)	5–15
Female sex parent	68	97.1		
Female sex child	23	32.9		
Parent education^1^				
High school or lower	3	4.3		
Intermediate vocational education	22	31.4		
University	44	62.9		
Marital status^1^				
Married/registered partnership	50	71.4		
Partner but unmarried	8	11.4		
Single	8	11.4		
Divorced	3	4.3		
Socio-cultural identity parent^1^				
Dutch	67	95.7		
Other	2	2.9		
**Descriptives**				
Parental Resilience			18.4 (4.3)	6–26
Parental Empowerment			48.0 (6.3)	27–61
Child Internalizing problems			17.5 (2.9)	11–24
Child Externalizing problems			14.1 (3.4)	7–22

Note. ^1^For these variables, *N* = 1 was missing

The Pearson correlations between all variables are shown in Table 2 (Table 2). Parental resilience was significantly positively correlated with parental empowerment (*r* =.49, *p* <.001). Age showed a significant negative correlation with externalizing problems (*r* = −.25, *p* =.046). None of the other correlations achieved significance.

**Table 2:** Correlation matrix

	1.	2.	3.	4.	5.	6.	7.
1. Resilience	1	0.49^**^	− 0.11	0.13	0.07	0.11	0.20
2. Empowerment		1	− 0.09	− 0.08	0.10	− 0.15	− 0.07
3. Internalizing problems			1	0.12	0.05	0.11	− 0.10
4. Externalizing problems				1	− 0.25^*^	− 0.09	− 0.04
5. Child age					1	0.01	− 0.10
6. Child sex						1	0.24
7. Education level							1

Note. ^**^p <.01. ^*^p <.05

### Hierarchical Regressions

The results of the hierarchical linear regressions can be found in Table 3 (Table 3). Regarding internalizing problems, the results revealed that model 1 did not account for a significant amount of variance in this outcome variable, *F* (2,62) = 0.43, *p* =.650, *R*^2^ = 0.01. Neither parental resilience (*β* = -0.09, *p* =.525), or parental empowerment (*β* = -0.04, *p* =.783) were significant predictors. The second model, including the covariates, did not account for a significant amount of variance in internalizing problems either (*F* (5,59) = 0.52, *p* =.762, *R*^2^ = 0.04). In this model, none of the predictors achieved significance. The variance in internalizing problems explained by model 2 compared to model 1 was not significantly different (∆*F* (3,59) = 0.58, *p =*.630, *∆R2* = 0.03).

With respect to externalizing problems, the results indicated that the first model did not significantly explain the variance in this outcome variable (*F* (2,62) = 1.39, *p* =.256, *R*^2^ = 0.04). In this model, neither resilience (*β* = 0.22, *p* =.130), nor empowerment (*β* = -0.19, *p* =.191) were significant predictors of externalizing problems, even though betas were approaching a medium effect size. The inclusion of covariates in the second model did not result in a significant explained variance in relation to externalizing problems (*F* (5,59) = 1.87, *p* =.114, R^2^ = 0.14). In addition, the variance in externalizing problems explained by model 2 compared to model 1 was not significantly different (∆*F* (3,59) = 2.13, *p =*.106, *∆R2* = 0.09).Yet, in this model both parental resilience (*β* = 0.29, *p* =.049) and child age (*β* = -0.26, *p* =.040) were significant predictors of externalizing problems. Child sex (*β* = -0.13, *p* =.321), parental education level (*β* = -0.12, *p* =.371) and parental empowerment (*β* = -0.23, *p* =.121) did not achieve significance.

**Table 3:** Hierarchical regression analysis for internalizing and externalizing problems

		Internalizing problems				Externalizing problems			
		B (SE)	β	t	*p*	B (SE)	β	t	*p*
Model 1	Resilience	-0.07 (0.10)	-0.09	-0.64	0.525	0.18 (0.12)	0.22	1.54	0.130
	Empowerment	-0.02 (0.07)	-0.04	-0.28	0.783	-0.10 (0.08)	-0.19	-1.32	0.191
Model 2	Child age	0.06 (0.15)	0.05	0.39	0.696	-0.35 (0.17)	-0.26	-2.10	0.040^*^
	Child sex	0.87 (0.83)	0.14	1.05	0.300	-0.91 (0.90)	-0.13	-1.00	0.321
	Parental education	-0.68(0.84)	-0.11	-0.81	0.423	-0.82 (0.92)	-0.12	-0.90	0.371
	Resilience	-0.07(0.11)	-0.10	-0.62	0.541	0.24 (0.12)	0.29	2.01	0.049^*^
	Empowerment	-0.01(0.07)	-0.03	-0.20	0.839	-0.12 (0.08)	-0.23	-1.57	0.121

Note. ^*^p <.05. *N* = 65

### Exploratory Analyses

#### Moderator Analysis

**Fig. 1:**
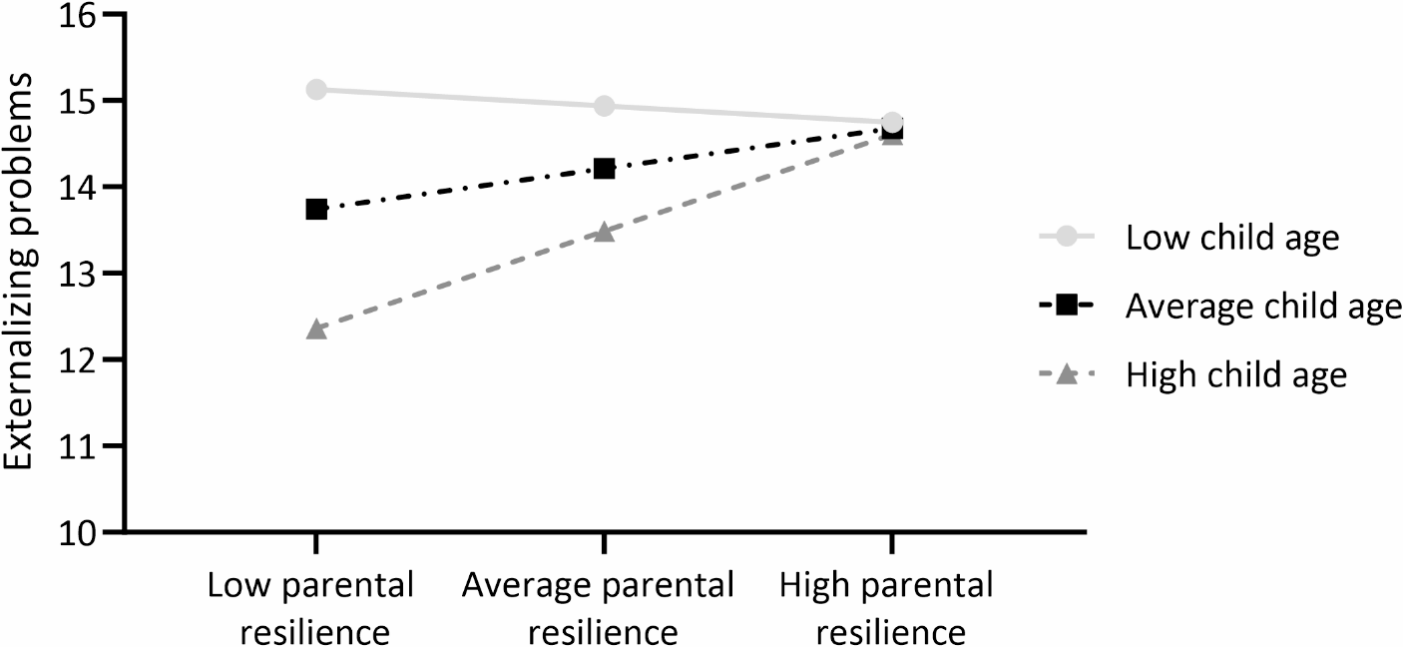
Moderation analysis with child age as moderator. *Note.* Low child age = 2.49 SD below average child age. Average child age = 11.1 years old. High child age = 2.49 above average child age

Based on the results of the hierarchical regressions, a moderation analysis in PROCESS was conducted, with parental resilience as the independent variable, child age as moderator and externalizing symptoms as the dependent variable. The analysis did not reveal a significant interaction effect between parental resilience and child age (*b* = 0.06, *SE* = 0.04, *t* = 1.42, *p* =.160). Neither child age (*b* = -1.40, *SE* = 0.76, *t* = -1.85, *p* =.069) nor parental resilience (*b* = -0.57, *SE* = 0.48, *t* = -1.19, *p* =.238) were significant predictors of externalizing problems in this model (*F* (3,62) = 2.47, *p* =.070, *R*^2^ = 0.11). Yet, when plotting child age, the lines of the different age groups cross, indicating an interaction effect in which the relation between parental resilience and externalizing symptoms changes depending on child age (Fig. 1). In older children, higher levels of parental resilience seem to be related to more externalizing symptoms, while the reverse seems true for younger children.

#### Kruskal-Wallis Test

Through post hoc analysis using the Kruskal-Wallis test, it was evaluated whether parental resilience, parental empowerment, internalizing, and externalizing symptoms differed by children with ASD, ADHD and with both diagnoses (Table 4). The results indicated that there were no significant differences between levels of parental resilience (*H* = 0.59, *p* =.746), empowerment (*H* = 0.11, *p* =.946), and internalizing symptoms (*H* = 3.08, *p* =.214) between the three groups. The groups did differ significantly regarding externalizing symptoms (*H* = 14.15, *p* <.001), with the ASD + ADHD group having the highest scores.

**Table 4:** Kruskal-Wallis test to compare diagnosis groups

	Group	N	Mean Rank
Parental resilience	ADHD	13	37.12
	ASD	37	32.51
	ASD + ADHD	17	34.85
Parental empowerment	ADHD	12	35.13
	ASD	37	33.26
	ASD + ADHD	17	32.88
Internalizing symptoms	ADHD	14	27.64
	ASD	38	35.39
	ASD + ADHD	17	40.18
Externalizing symptoms	ADHD	14	39.64
	ASD	38	27.28
	ASD + ADHD	17	48.44

## Discussion

The current study aimed to examine the associations between parental empowerment and resilience, and the degree of internalizing and externalizing behaviors in children with ASD and/or ADHD. Preliminary regression results revealed that higher levels of parental resilience were associated with more externalizing problems, unmasked by controlling for child age, child sex, and parental education level. Furthermore, older children were reported to have fewer externalizing symptoms. Yet, moderation analysis did not reveal the relation between resilience and externalizing behaviors to be significantly different according to child age. Nevertheless, the pattern observed in the data suggested a nuanced relation; in younger children, higher levels of resilience seemed to be linked to fewer externalizing behaviors, while in older children, resilience was related to more externalizing symptoms. This observed interaction effect of age, while not statistically significant, could potentially be attributed to the limited sample size.

Contrary to our expectations, parental resilience was related to higher levels of externalizing behaviors in children. This association emerged after controlling for parental education level, child age, and child sex, which were all negatively related to externalizing behaviors. It is of note that though the covariate model was more precise, it did not explain a significant amount of variance. Furthermore, there is limited research into this relation, especially in neurodivergent children, making it difficult to compare our findings to existing literature. Nevertheless, the results align with the concept of parental resilience as a process of adaptation (Gavidia-Payne et al., 2015; Masten, 2001). Resilience, within this context, refers to the competence demonstrated by parents in using skills, strengths, and knowledge to adaptively handle the challenges of parenting. This competence can strengthen with experience (Gavidia-Payne et al., 2015). Current findings revealed that parents who reported more externalizing symptoms in their children with ASD and ADHD also self-reported higher resilience scores. This may suggest that parents of children with elevated levels of externalizing symptoms have more experience in managing these symptoms and thus might develop greater resilience. This may imply that resilience is not solely an outcome of raising a neurodivergent child, but also a mechanism for adapting to the increased parenting demands. To better understand this relation, future research should test this hypothesis using a longitudinal study design, which allows for capturing changes in both resilience and externalizing symptoms over time. Such a longitudinal study might also gain insights in whether parental resilience is a key aspect of, or contributes to, the broader concept of parent gain (i.e., parental personal growth) associated with caring for a child with ASD or ADHD (Weiss et al., 2015). The current findings suggest that among other examples, such as increased compassion and patience, parent gain in parents of children with ASD or ADHD may also include higher levels of parental resilience. Practically, the present findings imply that interventions aimed at reducing problem behaviors in neurodivergent children should also focus on supporting parental skills for child behavioral management (e.g., positive reinforcement or limit-setting techniques) to boost parental resilience (Prevedini et al., 2020).

Contrary to our hypothesis, parental empowerment was not found to be a significant predictor of child internalizing or externalizing symptoms. Most existing research into this relation is treatment-focused and conducted in children with behavioral problems (Damen et al., 2019, 2021). These studies, conducted at youth care organizations, indicated that improvements in parental empowerment from pre- to post-treatment (i.e., both child-centered and family-centered treatments) were related to fewer behavioral problems in children (Damen et al., 2019). However, the results of the current study suggest that parental empowerment may not serve as a direct predictor of child outcomes in neurodivergent children. As previous research suggests, parental empowerment may serve as a mediator or moderator in the relation between child problem behaviors and parental distress (Damen et al., 2021; Weiss et al., 2015). While Damen et al. (2021) found that parental empowerment moderated the relation between parental stress and child behavioral problems, the results of Weiss et al. (2015) supported a partial mediating role of empowerment in the relation between greater problem behavior in children with ASD and maternal distress. Thus, future studies should further investigate the role of parental empowerment in relation to child problem behaviors and parental distress.

The current study did not find any relation between parental protective factors and internalizing symptoms in children diagnosed with ADHD and/or ASD. Generally, externalizing symptoms are more noticeable to parents and cause more friction in family life, leading to higher reporting frequencies (Bein et al., 2015; De Los Reyes & Kazdin, 2005). Internalizing problems, on the other hand, might be less apparent to parents. This might lead to more discrepancy between parent report and child experience of internalizing problems (Bein et al., 2015). Additionally, the timing of data collection, specifically in relation to child age, may play a role. While depressive symptoms tend to increase later in adolescence, anxiety symptoms are more common during childhood (< 10 years old) (Gonzales et al., 2011). Given that the mean age of children in the current study was 11, it is possible that the study did not capture peak periods of internalizing symptoms. Similarly, in younger, neurotypical children (age 5), previous research did find a negative relation between positive maternal mental health (e.g., self-efficacy, self-esteem) and both externalizing and internalizing symptoms (Clayborne et al., 2023). In neurodivergent children, previous research has often combined internalizing and externalizing problems into a single category of “problem behaviors” or focused solely on externalizing behaviors, such as conduct problems (Aydin, 2023; Falk & Lee, 2012). The current findings thus support the idea that internalizing and externalizing problems should be distinguished when investigating the relation between parental factors and child outcomes. Combining these problems into a total problem score is less valuable, as they seem to be influenced differently by parental factors.

Regarding the covariates, only child age significantly predicted child problems, with older children having fewer externalizing symptoms. These results are in line with prior literature, indicating that most neurotypical children show a decrease in externalizing symptoms from early childhood to adolescence (Roskam, 2019). As in the current study, previous research has found this predictive effect of age only for externalizing symptoms and not for internalizing symptoms (Silverman et al., 2022). Therefore, child age might be an important confounder in the relation between parental protective factors and child externalizing symptoms. The results of the moderation analysis hinted that specifically in older children, higher levels of parental resilience were related to more child externalizing problems. In contrast, resilience seemed to be associated with less externalizing symptoms in younger children. This implies that younger children, who on average present more challenging behaviors, have parents who report lower resilience in managing these challenges. Conversely, parents of older children with still persistent externalizing symptoms may demonstrate greater resilience, as they may be better equipped to cope with these challenges. The apparent interaction effect of age, though not statistically significant, might reflect the small sample size. Therefore, these results should be interpreted with caution and warrant future investigation into the role of child age in the relation between parental factors and child behavioral problems.

The current study has several limitations that need to be acknowledged. Firstly, the sample size was too small to provide sufficient statistical power, which reduces the ability to detect significant effects and limits the generalizability of the findings. Generalizability was also limited because participants were predominantly highly educated Dutch mothers who were mostly married. Thus, our findings may not generalize to families with greater socioeconomic or ethnic diversity, or to fathers. Future research would thus benefit from including a larger and more diverse sample, incorporating assessments of both parents. Second, we did not include variables relating to parental distress. Previous research has shown that parental protective factors, such as empowerment and optimism, might work as a moderator or mediator in the relation between parental distress and behavioral problems in both neurodivergent and neurotypical children (Cabrera et al., 2021; Damen et al., 2021; Seely & Mickelson, 2019; Weiss et al., 2015). Thus, for future studies investigating the relation between parental protective factors and child problems, it would be beneficial to include an assessment of parental distress to examine the interplay between this risk factor and potential protective factors. This is especially relevant as parents of neurodivergent children often report higher stress levels (Craig et al., 2016; Giovagnoli et al., 2015). In addition, future research might consider examining the mediating role of parenting practices in the relation between parental protective factors and child outcomes (Evans et al., 2020; McRae et al., 2019). Thirdly, data relied on parent-reported assessments for their own resilience and empowerment levels and their child’s externalizing and internalizing symptoms. As a result, reporting bias cannot be ruled out. Furthermore, the questionnaire used to assess parental resilience (i.e., the BRS) was short and assessed general resilience rather than parental resilience. Thus, future research could examine whether there are differences between general resilience and specifically parenting resilience (e.g., Parenting Resilience Elements Questionnaire (Suzuki et al., 2015) in relation to child behavioral problems. Despite these limitations, the current study addresses a significant research gap by focusing on two important parental protective factors which are highly understudied, especially in neurodivergent children. Consequently, this study moves away from the unbalanced focus on parental risk factors and deficits.

In sum, the current study provides preliminary insights into the associations between parental protective factors and child internalizing and externalizing behaviors in ASD and ADHD by showing that both parental resilience and child age were predictive of child externalizing problems. The positive relation between parental resilience and externalizing symptoms suggests that resilience might be a mechanism for adapting to increased parenting demands associated with raising a neurodivergent child with problem behaviors. Regarding parental empowerment, future research might explore the role of this protective factor as a potential mediator or moderator in the relation between child problem behaviors and parental distress. Lastly, the data suggest potential age-related differences in how parental resilience impacts child behavior, which highlights the need for future investigation with a larger and more diverse sample.

## Data Availability

The participants of this study did not give written consent for their data to be shared publicly, so due to the sensitive nature of the research supporting data is not available.
